# Editorial: Emerging talents in neuromorphic engineering

**DOI:** 10.3389/fnins.2024.1386712

**Published:** 2024-02-28

**Authors:** Horacio Rostro-Gonzalez, Juan Pedro Dominguez-Morales, Bernard Girau, Fernando Perez-Peña

**Affiliations:** ^1^GEPI Research Group, School of Engineering - IQS, Universitat Ramon Llull, Barcelona, Spain; ^2^Robotics and Technology of Computers Lab (RTC), ETSI Informática, Universidad de Sevilla, Sevilla, Spain; ^3^Laboratoire Lorrain de Recherche en Informatique et ses Applications, Centre National de la Recherche Scientifique, Université de Lorraine, Nancy, France; ^4^Department of Automation, Electronics and Computing Architecture and Networks, University of Cadiz, Puerto Real, Spain

**Keywords:** neuromorphic engineering, event-based image sensor, Spiking Neural Network (SNN), gradient-based method, neuro-inspired computing, Loihi, oscillatory neural network

This Research Topic provides a platform to highlight the outstanding contributions of emerging talents in the field of neuromorphic engineering. Through this dedicated series, we aim to showcase the promising work of student researchers within Neuromorphic Engineering.

The first article of this Topic Purohit and Manohar introduces a novel approach to address the limitations of conventional frame-based image sensors and event-based image sensors. While frame-based sensors suffer from high bandwidth requirements and power consumption, event-based sensors face challenges related to latency and timing errors as the number of pixels with an event increases. The proposed solution, termed Field-Programmable AER (FP-AER) encoding scheme, combines the advantages of both frame-based and event-based approaches. FP-AER allows for “in the field” configuration using configuration bits, offering flexibility and adaptability. The article evaluates the performance of FP-AER against existing AER-based approaches for imaging applications, demonstrating superior performance in both scanning and event-based readout scenarios.

In Bitar et al., authors explore the adaptation and evaluation of gradient-based explainability methods for Spiking Neural Networks (SNNs), aiming to shed light on how these networks process information. While SNNs mimic biological neurons and offer advantages such as ultra-low latency and small power consumption, existing explainability methods for SNNs are limited in scalability and effectiveness. The adapted methods in this study address these limitations by creating input feature attribution maps for SNNs trained through backpropagation, allowing for the identification of highly contributing pixels and spikes. Evaluation on classification tasks for both real-valued and spiking data confirms the accuracy of the proposed methods, showcasing their potential to enhance our understanding of SNNs and contribute to the development of more efficient networks.

The study presented in Dey and Dimitrov delves into the validation and testing of emerging computational architectures in the realm of neuro-inspired computing. Dividing the validation process into two key phases, the research first establishes a methodological and numerical framework for comparing neuromorphic and conventional platforms. Leveraging the Leaky Integrate and Fire (LIF) model based on data from the mouse visual cortex, where neuromorphic chip Loihi is employed as the test platform, with results validated against classical simulations. Demonstrating efficient replication of classical simulations with high precision on Loihi, the study proceeds to a sensitivity analysis, assessing the robustness of the model regime by varying significant parameters. Through meticulous assessment of single and dual parameter changes, the research identifies robustness in the majority of parameters, while pinpointing specific parameters requiring greater precision definition for heightened sensitivity.

Finally, in Jiménez et al. authors explore alternative paradigms to the von Neumann computing scheme gain traction, such as oscillatory neural networks (ONNs) utilizing phase-change materials like VO2. These ONNs offer an energy-efficient, massively parallel, brain-inspired, in-memory computing approach by encoding information in the phase pattern of frequency-locked oscillators. Despite the widespread adoption of the Hebbian learning rule for configuring ONNs, alternative learning algorithms have shown superior performance in Hopfield networks. However, not all of these algorithms are applicable to ONN training due to physical implementation constraints. In this regard, authors evaluate various learning methods for their suitability in ONNs, proposing a new approach that demonstrates competitive results in pattern recognition accuracy with reduced precision in synaptic weights, and is suitable for online learning when compared to previous works.

We trust that this Research Topic will serve as a valuable reference for exploring the current advancements in tools grounded in information theory and their application to neuroscience, providing insightful insights into this emerging field, particularly within the realm of neuromorphic engineering.

## Author contributions

HR-G: Writing—original draft, Writing—review & editing. JD-M: Writing—original draft, Writing—review & editing. BG: Writing—original draft, Writing—review & editing. FP-P: Writing—original draft, Writing—review & editing.

